# O-54 HOW DO WE KNOW A HEALTH PROTECTION SYSTEM PROTECTS THE POPULATION? USING THE HEALTH PROTECTION ASSURANCE FRAMEWORK TO SEEK CLARITY, ACCOUNTABILITY AND SYSTEM WIDE IMPROVEMENT

**DOI:** 10.1093/pubmed/fdag046.048

**Published:** 2026-07-09

**Authors:** Miss Nadine Hilliard, Mrs Ania Hewis, Mr David Pearce, Mr Mike Sandys, Dr Jessica Banks

**Affiliations:** UK Health Security Agency (UKHSA); UK Health Security Agency (UKHSA); UK Health Security Agency (UKHSA); Leicestershire County Council; Derbyshire County Council

## Abstract

**Background:**

Health Protection systems (HPs) are complex, multi-agency networks requiring effective cross-sectoral collaboration to protect population health. Launched in 2024, the Health Protection Assurance Framework (HPAF) was developed as a robust, flexible tool to convene professionals across organisational boundaries and stimulate structured dialogue regarding local health protection arrangements. It supports Directors of Public Health in fulfilling their statutory duty to ensure adequate system-wide protections are in place.

**Objectives:**

One year following implementation, a qualitative evaluation was conducted using the Consolidated Framework for Implementation Research (CFIR) to explore the integration and outputs of the HPAF across HPs in the East Midlands.

**Methods:**

Semi-structured interviews were conducted with representatives from all ten Upper Tier Local Authority in the East Midlands. Data was analysed using framework analysis supported by the CFIR to explore implementation models, contextual factors, barriers, facilitators and system impact.

**Results:**

All local HPs had implemented the HPAF and it had been adapted to align with local ways of working and supported HPs to achieve a shared view of the strengths, gaps and priorities. The framework had a consistently positive impact on assurance processes regardless of the implementation approach.

Key themes identified included:

- Improved oversight and transparency of system gaps, such as antimicrobial resistance.
- Enhanced partner communication via a shared template for consistent messaging.
- Strengthened governance by clarifying roles and accountabilities across the system.
- Provision of a structured evidence base to justify priorities and maintain continuity during political or structural transitions.

**Conclusions:**

The evaluation has identified that utilising the HPAF has offered clear benefits for HPs; it formalised assurance processes and acted as a strategic resource for facilitating collaboration and continuity within increasingly pressured HPs. Its collaborative design provides a strong foundation for ongoing quality improvement and system resilience and demonstrates how shared frameworks can support more effective and integrated HPs.

